# The Prevalence and Associated Risk Factors of Coronary Heart Disease in Patients With Gout: A Cross-Sectional Study

**DOI:** 10.7759/cureus.85194

**Published:** 2025-06-01

**Authors:** Sohaib Asghar, Shoaib Asghar, Summayha Mahbub, Asad Riaz, Shahid Farooq, Imran Khan

**Affiliations:** 1 Cardiology, Morriston Hospital, Swansea Bay University Health Board, Swansea, GBR; 2 Internal Medicine, Sheikh Zayed Medical College/Hospital, Rahim Yar Khan, PAK; 3 Haematology, Singleton Hospital, Swansea Bay University Health Board, Swansea, GBR; 4 Medicine, East Kent Hospital University Foundation Trust, Ashford, GBR; 5 Gastroenterology, Peterborough City Hospital North West Anglia NHS Trust, Peterborough, GBR; 6 Internal Medicine, Services Hospital Lahore, Lahore, PAK

**Keywords:** allopurinol, coronary heart disease (chd), gout, gouty tophi, hypercholesterolemia, hypertension, nonsteroidal anti-inflammatory drugs (nsaids), obesity, risk factors for cardiovascular diseases, type 2 diabetes mellitus(t2dm)

## Abstract

Background

Long-term gout patients have been linked to increased risk of coronary heart disease (CHD) and its associated comorbidities. Therefore, careful evaluations are necessary to examine the prevalence and the causes of CHD in gout patients. The aim of this research was to identify significant factors associated with CHD in patients with gout along with the prevalence rate.

Materials and methods

A cross-sectional study was performed on 270 gout patients who attended the specialist outpatient clinic and the medicine department of Sheikh Zayed Medical College & Hospital, Rahim Yar Khan, Pakistan. Data about predictor variables was collected through interviews and standard clinical procedures. Blood samples were taken to measure laboratory variables such as fasting blood sugar (FBS), serum creatinine, cholesterol, triglycerides, high-density lipoprotein cholesterol (HDL-C) and low-density lipoprotein cholesterol (LDL-C). Initially, descriptive statistics along with the chi-square test of association was executed. Finally, logistic regression analysis was performed as a classification method to estimate the odds ratio and confidence interval.

Results

The findings showed that 108 (40%, n=270) of gout patients experienced CHD with the highest prevalence rate (52; 48%, n1=108) of myocardial infarction (MI). Among all (n=270) patients, 150 (56%) were suffering from gout for more than 10 years, and 161 (60%) were diagnosed with hypertension. Considering p-value<0.05 as significant for the chi-square test, hypertension (p-value=0.001), type 2 diabetes mellitus (T2DM) (p-value=0.004), family history of CHD (p-value=0.001), serum creatinine (p-value=0.02), cholesterol (p-value=0.04), subcutaneous tophi (p-value=0.02), intraosseous tophi (p-value=0.04), and intermittent allopurinol intake (p-value=0.04) were found to be associated with CHD in patients with gout. The logistic regression analysis showed that hypertension (OR=2.70, CI: 1.60-4.58), T2DM (OR=2.08, CI: 1.26-3.42), creatinine (OR=1.77, CI: 1.07-2.93), cholesterol (OR=1.67, CI: 1.02-2.73), HDL-C (OR=0.59, CI: 0.36-0.96), subcutaneous tophi (OR=1.86, CI: 1.09-3.16), intraosseous tophi (OR=2.22, CI: 1.29-3.81), and intermittent allopurinol consumption (OR=1.92, CI: 1.01-3.66) were associated with CHD in patients with gout.

Conclusions

The study estimated that 40% of patients with chronic gout developed different types of CHD with the highest prevalence of MI, regardless of the duration of gout or treatment of acute attacks. Hypertension, T2DM, obesity, high creatinine, high cholesterol, low HDL-C and presence of intraosseous tophi are found to be significant factors of CHD in gout patients. These findings significantly contributed to medical research to address the risk of CHD among Pakistani patients with gout by controlling these significant associated factors. Gout patients with a positive family history of CHD should also take precautionary measures to avoid heart complications. Nation-wide large-scale analyses considering rural/urban disparities are recommended for more in-depth investigations of influencing factors to prevent cardiovascular complications among patients with gout since diversity in demographic, lifestyle and environmental factors may influence the overall impact. Future analysis with lipids as a continuous variable is also suggested for more accurate estimates.

## Introduction

Gout, an inflammatory disease, is the most frequently reported arthritis due to modified dietary habits, lifestyle manners and environmental components [1,2]. Frequent gout symptoms are severe pain, swelling, redness of joints, intermittent gout attacks and limited joint movement. Some other related signs may include high uric acid, fatigue, high fever and tophi in critical conditions [3]. Stages of gout primarily include hyperuricemia, secondly gout flares, then intercritical gout and finally tophi. After several years, untreated hyperuricemia leads to tophi which can damage joints, soft tissues and kidneys [4]. 

Gout is completely treatable in most cases by using proper treatments, timely diagnosis and improved lifestyle. The reported significant factors of gout are hyperuricemia, positive family history, dietary habits, hypertension, chronic kidney disease, obesity and alcohol intake [2,5]. Notably, a low standard of living is highly associated with gout by several scientific research studies in the literature [6-8]. Gout is an independent predictor of diabetes mellitus, obesity, nephrolithiasis, hypertension and other kidney diseases. Several studies identified that patients with gout are at higher risk of cardiovascular morbidity like hypertension, myocardial infarction, heart attack, cardiac dysrhythmia, ischemic or hemorrhagic stroke and peripheral vascular disease. It is reported that even after controlling other risk factors, gout patients independently have a higher risk of cardiovascular mortality [2,9,10].

Coronary heart disease (CHD) occurs when the coronary arteries, which supply blood to the heart, become narrowed or blocked due to atherosclerosis, restricting blood flow to the heart muscle. This reduced blood flow can cause symptoms such as angina and, in severe cases, can lead to a myocardial infarction (MI) due to complete blockage of a coronary artery. Gout patients have a higher risk of developing CHD due to chronic inflammation, hyperuricemia, kidney disease, hypertension, and metabolic syndrome. A significantly strong association of gout with the development of cardiovascular diseases is established in addition to the increasing rate of comorbidities and duration of gout [11,12].

The pathophysiological interplay between CHD and gout originates in their mutual mechanisms regarding inflammation and metabolic disturbances. Gout triggers intense inflammatory responses [10,11]. This inflammation can intensify atherosclerosis which is a vital feature of CHD. Atherosclerosis is increased due to high endothelial dysfunction, enhanced platelet activation, and promoted pro-inflammatory cytokine production [11,12]. In contrast, CHD's associated oxidative stress and impaired renal function can boost uric acid levels, further influencing individuals to gout attacks [13]. The interdependence between these disorders highlights the necessity of addressing both CHD and gout through multifaceted strategies focusing on inflammation, metabolic function, and cardiovascular risk factors [11-13].

Each gout stage is associated with CHD differently [11,13]. Asymptomatic hyperuricemia refers to elevated uric acid levels without symptoms, which may increase cardiovascular risk by promoting inflammation and impairing endothelial function, potentially leading to vascular damage and cardiovascular disease [13]. During acute gout arthritis, intense inflammatory responses can temporarily heighten cardiovascular risk by triggering systemic inflammation, which may lead to vascular instability, thrombosis, or cardiac stress [11,13,14]. During the intercritical phase of gout (between acute attacks), persistent chronic inflammation and elevated uric acid levels may contribute to ongoing cardiovascular risk, potentially leading to vascular damage and increased risk of cardiovascular events. In chronic tophaceous gout, the presence of tophi (urate deposits) indicates advanced disease. This stage is associated with increased cardiovascular risk due to chronic systemic inflammation from persistent urate deposition, kidney damage or impairment from prolonged hyperuricemia, and higher likelihood of cardiovascular comorbidities, such as hypertension and heart disease [11,14]. These factors can collectively contribute to a higher risk of cardiovascular events in patients with chronic tophaceous gout [4,11]. For the treatment of gout, the intake of allopurinol [13] and non-steroidal anti-inflammatory drugs (NSAIDs) [14] specifically prolonged use or heavy doses are reported as an increased risk of cardiovascular diseases at high doses or with long-term use, are associated with increased CVD risk, particularly in patients with pre-existing cardiovascular conditions. Duration, type and dose of gout medications, along with individual features of patients, influence the overall effect.

Several past studies examined the risk factors and medical complications of gout. However, only a few studies have estimated the rate of occurrence of CHD in gout patients and identified significant predictors related to CHD in gout patients.

Markelova et al. [15] conducted a study in Moscow to examine the prevalence and factors of CHD in gout patients using a logistic regression model. Regarding prevalence, gout patients diagnosed with CHD belonged to the older age group with a longer duration of gout and had a high percentage of intraosseous tophi and nephrolithiasis compared to the reference group. The study concluded that age, duration of gout and acute gout attack, intraosseous tophi, nephrolithiasis, chronic kidney disease, serum creatinine and continuous NSAID intake are significantly associated with CHD in male gout patients based on odds ratios [15,16]. De Vera et al. [17] conducted a study to examine the independent association between gout and acute myocardial infarction (AMI) in older women using the British Columbia Linked Health Database. Using the Cox regression model, the findings suggested that women with gout are at higher risk of AMI and even the risk is higher than men [17]. A cross-sectional investigation is conducted to examine the prevalence and risk factors of gout in the Pakistani population. The findings showed that hypertension is an associated risk factor of gout [18].

Extensive literature investigated CHD as a risk factor of gout for populations with regional and wealth disparities but only a few accounted for both disorders simultaneously. More comprehensive investigations are essential to identify risk factors of CHD in gout patients. Additionally, no past study has been conducted to investigate this subject for the Pakistani population. Some reasons for this deficiency may be diverse population features, limited applicability of findings, healthcare facilitation and policy concerns. Considering these shortcomings, this study primarily aims to examine the prevalence of CHD in patients with gout using recent data. Secondly, the significant factors of CHD are determined in Pakistani patients with gout. A cross-sectional design is used as it estimates the prevalence and identifies the associations simultaneously.

## Materials and methods

Operational definitions

Nephrolithiasis is the process of formation of stones within the kidneys. Subcutaneous tophi are lumps that form under the skin and intraosseous tophi refer to lumps within the bone due to the accumulation of urate crystals. Intermittent allopurinol intake refers to taking the allopurinol irregularly or in a non-continuous pattern, rather than as prescribed consistently to treat chronic gout. Continuous NSAID intake refers to the regular, ongoing use over a long period for acute gout attacks. NSAIDs for treating acute gout mean a short-term use to relieve pain and inflammation during a gout flare-up. Without NSAIDs refers to non-use of NSAIDs for gout.

Study design

This cross-sectional study was conducted from May 2021 to February 2023 on 270 gout patients who attended the specialist outpatient clinic and the medicine department of Sheikh Zayed Medical College & Hospital, Rahim Yar Khan, Pakistan. The study rationale was explained, informed consent was obtained and all patient biodata were kept confidential. 

The diagnosis of CHD was confirmed on the basis of complaints like angina pectoris, arrhythmia, heart failure, heart valve abnormality and MI by a cardiologist. Cardiologists used routine clinical methods including ECG (electrocardiogram), chest X-ray, echocardiography, cardiac biomarkers and other invasive imaging (like coronary angiography) to diagnose these patients with CHD on an outpatient basis.

Gout patients between the ages of 20 to 65 years who had had gout for more than five years were included in the study. Gout tophi were clinically diagnosed based on physical examination and confirmed with X-ray imaging for their types. This study excluded newly diagnosed gout patients, pregnant women, those with under five years of gout duration, those with age under 20 years or over 65 years, those with a low BMI of less than 20 kg/m², alcoholics, those with chronic liver disease and chronic kidney disease with creatinine greater than 3mg/dl, those with incomplete medical records and those with a history of poor medication adherence [15]. Low BMI was not included in the analysis due to the lack of substantial evidence in the literature supporting a significant association between low BMI and the risk of gout. This study also excluded patients with joint problems and deformities caused by any connective tissue disorder or rheumatological conditions such as rheumatoid arthritis, seronegative spondyloarthropathies like ankylosing spondylitis, psoriatic arthritis, reactive arthritis, and inflammatory bowel disease (IBD)-associated arthritis.

Data collection

Demographic and clinical baseline characteristics were collected through patient interviews. The convenient sampling method is adopted. A power analysis with 80% desired power and 0.05 level of significance is conducted to calculate the sample size. All gout patients were examined for risk factors associated with the development of heart diseases such as age (20-50 years/ >50 years), gender (male/female), obesity (yes/no), BMI (20-29.9 kg/m²/ ≥30 kg/m²), hypertension (yes/no), T2DM (yes/no), smoking (yes/no), and family history of CHD (yes/no) [15]. BMI was computed by dividing weight in kilograms by height in meters squared (kg/m²). Blood pressure was measured on the right arm while seated using a mercury manometer. Obesity is measured based on waist circumference and clinical diagnosis.

Laboratory variables were analysed from a blood sample. The levels of creatinine were categorized as <1.30 mg/dl (normal) and ≥1.30 mg/dl (impaired), cholesterol range was divided into <200 mg/dl (normal) and ≥200 mg/dl (impaired), triglycerides were classified as <150 mg/dl (normal) and ≥150 mg/dl (impaired) [15]. The LDL-C level was binary categorised as <130 mg/dl (normal) and ≥130mg/dl (impaired), and HDL-C was grouped as <40 mg/dl (impaired) and ≥40 mg/dl (normal). Other observed factors included subcutaneous tophi (yes/no), intraosseous tophi (yes/no), nephrolithiasis (yes/no), continuous intake of allopurinol (yes/no), consumption intermittent allopurinol (yes/no), continuous intake of NSAIDs (yes/no), NSAIDs for treating acute gout (yes/no), and without NSAIDs (yes/no) [15]. Some variables measured on a continuous scale were categorised due to specified clinical relevance, easy understanding and interpretation, and standard risk assessment. Any observation with missing data was not entertained.

Data analysis

Statistical analysis was performed using statistical software IBM SPSS Statistics for Windows, Version 21 (Released 2012; IBM Corp., Armonk, New York, United States). Descriptive statistics including frequency and percentage were reported. The patients with gout were categorised into two classes depending on the occurrence and non-occurrence of CHD: Class I featuring gout patients diagnosed with CHD, and Class II having gout patients without a diagnosis of CHD. The chi-square test of association was performed to examine the statistically significant difference between the two classes at a p-value<0.05. Grouping of gout patients was considered as the response variable having values “1” for Class-I and “0” for Class-II. Finally, univariate and multivariate logistic regression model was executed for computing the odds ratio (OR) and 95% confidence interval (CI) to identify the important predictor variables of CHD in gout patients. The forest plot was further constructed for visual display of significant estimates obtained by multivariate logistic regression.

## Results

This study identified 108 (40%) gout patients with CHD and 162 (60%) gout patients without any diagnosis of CHD. The mean ± standard deviation (SD) is computed for continuous predictor variables. For Class I (Gout+CHD), the mean±SD age was 52.3 ± 11.5 years, the duration of gout was 8.3 ± 4.2 years and the age of gout onset was 42.7 ± 4.3 years. The duration of the last acute gout flare was 3.2 weeks with an arthritis frequency of 3 per year. The mean±SD BMI was 28.2 ± 5.5 for Class I (Gout+CHD) and 25.9 ± 6.1 for Class II (Gout-CHD). The distribution of baseline characteristics according to CHD is shown in Table 1.

**Table 1:** Distribution of baseline characteristics according to gout diagnosis with/without coronary heart disease (n=270) CHD, coronary heart disease; BMI, body mass index; T2DM, type 2 diabetes mellitus Significant p-values of less than 0.05 are indicated in bold

Factors			Cross-tabulation and Chi-Square Test			
Total (N)= 270		Prevalence (%)	Class I (Gout + CHD) (n1=108)	Class II (Gout – CHD) (n2=162)	Pearson Chi-square value	p-value
Age (Years)	20-50	147(54)	62	85	0.637	0.425
	>50	123(46)	46	77		
Gender	Male	138(51)	59	79	0.892	0.345
	Female	132(49)	49	83		
BMI kg/m²	20-29.9	145(54)	59	86	0.062	0.803
	≥30	125(46)	49	76		
Obesity	Yes	114(42)	42	72	0.820	0.365
	No	156(58)	66	90		
Duration of Gout (Years)	5-10	120(44)	44	76	1.00	0.317
	>10	150(56)	64	86		
Hypertension (>140/90 mmHg)	Yes	161(60)	49	112	15.203	0.001
	No	109(40)	59	50		
T2DM	Yes	109(40)	55	54	8.331	0.004
	No	161(60)	53	108		
Smoking	Yes	62(23)	27	35	0.422	0.516
	No	208(77)	81	127		
Family History	Yes	87(32)	48	39	12.312	0.001
	No	183(68)	60	123		

The findings in Table 1 showed the distribution of basic characteristics and p-values of the chi-square test of association for Class I and Class II patients. The chi-square test of association showed a significant difference of hypertension, T2DM, and family history of CHD between two classes of gout patients. Gender, age, BMI, obesity, duration of gout, and smoking are observed to be non-significant variables for the observed data.

Table 2 shows the frequency distribution, cross-tabulation, and p-value based on the chi-square test of association over a dataset of gout patients. 

**Table 2:** Distribution of clinical factors according to gout diagnosis with/without coronary heart disease (n=270) CHD, coronary heart disease; LDL-C: low-density lipoprotein cholesterol; HDL-C: high-density lipoprotein cholesterol; NSAIDs, nonsteroidal anti-inflammatory drugs Significant p-values of less than 0.05 are indicated in bold

Factors			Cross-tabulation and Chi-Square Test			
Total (N)= 270		Prevalence (%)	Class I (Gout + CHD) (n1=108)	Class II (Gout – CHD) (n2=162)	Pearson Chi-square value	p-value
Creatinine (mg/dl)	<1.30	167(62)	58	109	5.065	0.02
	≥1.30	103(38)	50	53		
Cholesterol (mg/dl)	<200	148(55)	51	97	4.189	0.04
	≥200	122(45)	57	65		
Triglycerides (mg/dl)	<150	110(41)	39	71	1.598	0.20
	≥150	160(59)	69	91		
LDL-C (mg/dl)	<130	96(36)	39	57	0.024	0.87
	≥130	174(64)	69	105		
HDL-C (mg/dl)	<40	126(47)	126	67	4.586	0.32
	≥40	144(53)	144	95		
Subcutaneous tophi	Yes	79(29)	40	39	5.261	0.02
	No	191(71)	68	123		
Intraosseous tophi	Yes	74(27)	40	34	8.389	0.04
	No	196(73)	68	128		
Nephrolithiasis	Yes	71(26)	34	37	2.497	0.11
	No	199(74)	74	125		
Continuous allopurinol intake	Yes	28(10)	14	14	1.302	0.25
	No	242(90)	94	148		
Intermittent allopurinol intake	Yes	45(17)	24	21	4.000	0.04
	No	225(83)	84	141		
Continuous NSAIDs intake	Yes	69(26)	34	35	3..323	0.07
	No	201(74)	74	127		
NSAIDs for treating acute gout	Yes	213(79)	82	131	0.949	0.33
	No	57(21)	26	31		
Without NSAIDs	Yes	56(21)	25	31	0.635	0.43
	No	214(79)	83	131		

Among all gout patients, the clinical variables including serum creatinine (higher than>1.30mg/dl), cholesterol level (higher than≥200mg/dl), subcutaneous tophi and intraosseous tophi, and intake of intermittent allopurinol were significantly associated with two classes of respondents. The levels of LDL-C, HDL-C, triglycerides, continuous allopurinol intake, nephrolithiasis, continuous NSAID intake, NSAIDs for treating acute gout and without NSAIDs are found to be non-significant factors of CHD for the present data set. Akaike information criterion (AIC) is used for model assessment. The model showed AIC as 324.96.

**Figure 1:**
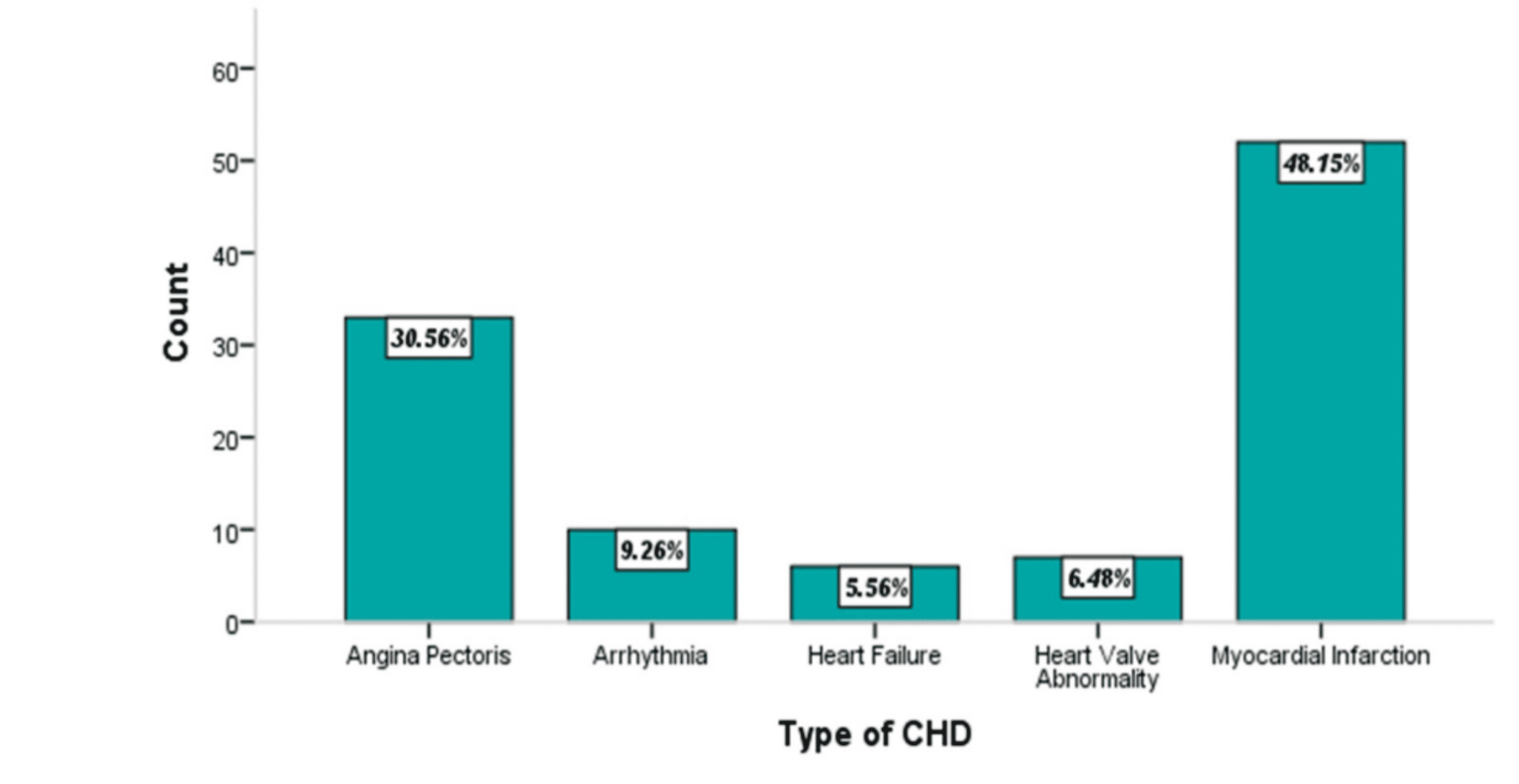
Bar plot showing the frequency and percentage distribution of types of coronary heart disease (n1=108) CHD, coronary heart disease

Figure 1 shows the observed types of CHD for Class I representing the highest percentage (52 patients; 48.15%) of MI followed by angina pectoris (33 patients; 30.56%), arrhythmia (10 patients; 9.26%), heart valve abnormality (7 patients; 6.48%) while only six (5.56%) gout patients experienced heart failure. 

Table 3 presents the classification analysis using a logistic regression model for binary response showing the crude odds ratio (cOR) obtained from univariate analysis and the adjusted odds ratio (aOR) computed from a multivariate model. 

**Table 3:** The univariate and multivariate logistic regression analysis for classification of gout diagnosis with and without coronary heart disease (n=270) OR, odds ratio; CI, confidence interval; BMI, body mass index; T2DM, type 2 diabetes mellitus *Significant p-values of less than 0.05 are indicated in bold

Variables	Univariate Logistic Regression		Multivariate Logistic Regression	
	cOR (CI 95%)	p-value	aOR (CI 95%)	p-value
Age (>50 years)	0.82 (0.50-1.34)	0.425	0.84(.44-1.61)	0.60
Gender (Male)	1.27(0.78-2.06)	0.345	1.18(0.54-2.58)	0.69
BMI (≥30kg/m²)	0.94(0.58-1.53)	0.803	0.85(0.43-1.70)	0.64
Obesity (Yes)	0.79(0.49-1.31)	0.366	0.39(0.19-0.81)	0.01*
Duration of Gout(>10 years)	1.29(0.79-2.10)	0.318	1.29(0.68-2.46)	0.44
Hypertension (Yes)	2.70(1.60-4.58)	0.000*	0.19(0.09-0.38)	0.001*
T2DM (Yes)	2.08(1.26-3.42)	0.004*	2.09(1.02-4.31)	0.45
Smoking (Yes)	1.21(0.68-2.15)	0.516	0.79(0.31-2.03)	0.63
Family History (Yes)	2.52(1.50-4.26)	0.001	2.69(1.28-5.66)	0.009
Creatinine (≥1.30mg/dl)	1.77(1.07-2.93)	0.025*	2.70(1.28-5.66)	0.01*
Cholesterol (≥200mg/dl)	1.67(1.02-2.73)	0.041*	1.89(0.99-3.61)	0.05*
Triglycerides (≥150mg/dl)	1.38(0.84-2.28)	0.207	1.75(0.90-3.40)	0.097
LDL-C (≥130mg/dl)	0.96(0.58-1.60)	0.876	0.70(0.37-1.33)	0.27
HDL-C (≥40mg/dl)	0.59(0.36-0.96)	0.033*	0.42(0.21-.084)	0.01*
Subcutaneous tophi (Yes)	1.86(1.09-3.16)	0.023*	1.42(0.59-3.40)	0.43
Intraosseous tophi (Yes)	2.22(1.29-3.81)	0.004*	4.04(1.67-9.81)	0.002*
Nephrolithiasis (Yes)	1.55(0.90-2.68)	0.115	0.87(0.31-2.44)	0.79
Continuous allopurinol intake (Yes)	1.57(0.72-3.45)	0.257	5.03(0.58-9.08)	0.14
Intermittent allopurinol intake (Yes)	1.92(1.01-3.66)	0.048*	4.57(0.80-6.27)	0.09
Continuous NSAIDs intake (Yes)	1.67(0.96-2.90)	0.07	0.27(0.05-1.29)	0.099
NSAIDs for treating acute gout (Yes)	0.75(0.41-1.35)	0.331	0.88(0.67-1.59)	0.89
Without NSAIDs (Yes)	1.27(0.70-2.31)	0.43	1.73(0.90-2.01)	0.62

Patients with gout were divided into two groups using the logit approach: those with and without CHD. The univariable logistic regression analysis showed that hypertension, T2DM, family history, creatinine, cholesterol, HDL-C, subcutaneous tophi, intraosseous tophi, and intermittent allopurinol intake significantly increased the model prediction. It is also observed that Class I patients (cOR=2.08) have two-fold greater odds of having T2DM than Class II respondents. Class I gout patients showed 2.52 times greater probability of belonging to a family with a positive history of CHD (OR=2.52) compared to Class II. High levels of creatinine (cOR=1.77) and cholesterol (cOR=1.67) increased 77% and 67% odds, respectively, of being in Class I compared to Class II. The odds of being in Class I are nearly 60% lower than Class II for respondents with higher levels of HDL-C (cOR=0.59). Subcutaneous tophi (cOR=1.86) and intraosseous tophi (cOR=2.22) increased the likelihood of existence by 86% and 122% respectively in Class I compared to Class II. The findings of univariate logistic regression showed that consumption of intermittent allopurinol for the treatment of gout (cOR=1.92) increased the odds of CHD (Class I) by 92%. 

The multivariate logistic regression analysis identified that obesity, family history of CHD, hypertension, creatinine, cholesterol, HDL-C, and intraosseous tophi are significantly associated with the occurrence of CHD in gout patients. The multivariate findings showed that obese gout patients have decreased odds of CHD compared to non-obese gout patients. This result for the current data may be the result of confounding.

**Figure 2:**
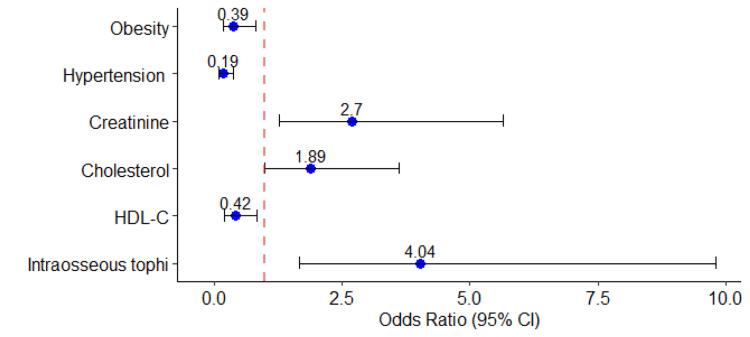
Forest plot showing significant predictors obtained from the multivariate binary logistic regression model HDL-C, high-density lipoprotein cholesterol

Figure 2 graphically displays the adjusted odds ratio (aOR) of significant variables with their confidence intervals obtained by executing a multivariate binary logistic regression model. 

The plot showed the association of these factors with Class I as creatinine and cholesterol have an aOR of greater than 1. The forest plot further showed the highest odds (OR=4.04) for intraosseous tophi showing the most influence of this predictor with the widest CI (1.67-9.81).

## Discussion

The present findings showed that hypertension, T2DM, family history of CHD, serum creatinine, cholesterol, subcutaneous tophi, intraosseous tophi, and intermittent allopurinol intake were found to be associated with CHD in patients with gout using the chi-square test of association. The logistic regression analysis showed that hypertension, T2DM, creatinine, cholesterol, HDL-C, subcutaneous tophi, intraosseous tophi, and intermittent allopurinol consumption were associated with CHD in patients with gout. These findings significantly contributed to medical research to address the risk of CHD among Pakistani patients with gout by controlling the associated factors.

A study by Mattiuzzi et al. [16] in 2020 on worldwide gout epidemiology reported that the comorbidities associated with gout may increase its mortality rate by more than 50% by 2060 [16]. Several population research studies, meta-analyses and comprehensive reviews indicated a high prevalence rate of CHD among gout patients, regardless of considering demographic, cultural, social and clinical features [1,16].

The present study found a significant prevalence rate (40%) of CHD in patients with gout for the observed dataset of Pakistan. Research by Markelova et al. [15] conducted in Moscow over 286 observations recorded 38.8% of gout patients diagnosed with CHD. Their study concluded that traditional risk factors of CHD, the gout severity and kidney failure history are associated with the development of CHD in patients with gout [15]. Consistent with the literature, the myocardial infarction (MI) rate is the highest form of CHD among Pakistani respondents with gout [12-14]. A population-based study [17] in British Columbia concluded that gout independently enhanced the probability of acute MI. This study by De Vera et al. [17] found that gouty elderly women had an elevated risk of MI and the magnitude of the excess risk is greater than in men. The mean age of the current sample was 52.3 years, with an average of 8.3 years of duration of gout and 3.2 weeks of the last acute gout flare. Markelova et al. considered the gout patients with a median age of 51.2 years having 6.2 years of gout duration [15].

The present findings showed that most respondents possessed a normal weight (156; 58%, n=270) and hypertension (161; 60%). A meta-analysis conducted in Canada by De Vera et al. [17] consistently reported that 65% of gout patients were hypertensive. Contrary to the recent findings over the observed data, abdominal obesity [15] was found in 95% of gout patients with CHD as weight control can prevent kidney disease and other associated complications.

A strong significant association of CHD with hypertension (p-value=0.001), T2DM (p-value=0.004), and family history of gout or comorbidities (p-value=0.001) is established among gout patients by the chi-square test of association. Several studies recommended the relationship of gout with the development of hypertension and T2DM [18-20]. Regarding smoking habits, an inverse association with gout is observed with the suggestion of its cessation to avoid the increased risk of cardiovascular diseases among patients with gout [21,22].

The findings obtained by the chi-square test showed that creatinine (p-value=0.02), cholesterol (p-value=0.04), subcutaneous tophi (p-value=0.02), intraosseous tophi (p-value=0.04), and intermittent allopurinol intake (p-value=0.04) are associated with CHD among gout patients. The studies by Roughley et al. [10], Markelova et al. [15] and Singh et al. [23] evidenced a noteworthy influence of chronic kidney disease and serum creatinine on CHD by using a dataset of patients with gout. The authors suggested mutually controlling both factors to decrease the probability of CHD in gouty persons [15,23].

Although the specific mechanism is not completely defined, gout is established as a significant risk factor for hypercholesterolemia as both disorders can be developed by metabolic conditions and living habits [2,24-26]. The Framingham Heart Study [24] reported that low HDL-C was the most compelling lipid predictor of CHD in men and women of greater than 49 years of age. The studies by Boden et al. [24] and Hashem et al. [25] signify that very low HDL-C may signify a non-linear inverse relationship with incident CHD risks.

Gouty tophi including subcutaneous, intraosseous and dermal tophi located in the joints, bones, and skin are observed by several previous studies [5, 9,13,18,19]. Further investigations reported tophi as an initial symptom of the syndrome although also found in chronic gout [19,26,27]. Longer duration of gout with joints and bones tophi increased risk of CHD in patients with intense gout [15,24,25].

The effectiveness and stability of consumption of intermittent allopurinol as initial treatment of gout are examined and verified [13,26]. A daily dose of 100-300 mg of allopurinol showed higher chances of regulating uric acid [26-28]. Additionally, the current binary logistic regression analysis showed the significant influence of obesity (p-value=0.01), family history of CHD (p-value=0.009) and HDL-C (p-value=0.01) on CDH among gout patients. Obesity is associated with the development of gout and CHD among gouty patients since weight gain links to hyperuricemia, fat, and disorder of metabolic functions [12,24-26,29-30]. 

The study included all gout patients aged between 20 and 65 years who had been diagnosed with gout for more than five years. This study excluded newly diagnosed gout patients, pregnant women, individuals under five years of gout duration, those under the age of 20 years or over the age of 65 years, individuals with a BMI of less than 20 kilograms per square meter (kg/m²), alcoholics, those with CHD, chronic liver disease, chronic kidney disease with a creatinine level greater than 3 milligrams per deciliter (mg/dl), incomplete medical records and a history of poor medication adherence [15]. This study also excluded patients with joint problems and deformities resulting from connective tissue disorders or rheumatological conditions, including rheumatoid arthritis, seronegative spondyloarthropathies such as ankylosing spondylitis, psoriatic arthritis, reactive arthritis and IBD-associated arthritis. Multiple chi-square tests were performed without applying correction methods, which increases the risk of type I errors. This limitation should be considered when interpreting the results.

## Conclusions

Gout is the most frequent arthritis globally with a high rate of mortality from CHD. This study estimated that 40% of patients with chronic gout developed different types of CHD with the highest prevalence of MI, regardless of the duration of gout or treatment of acute attacks. Hypertension, T2DM, obesity, high creatinine, high cholesterol, low HDL-C and presence of intraosseous tophi are found to be significant factors of CHD in gout patients. These findings significantly contributed to medical research to address the risk of CHD among Pakistani patients with gout by controlling these significant associated factors. Gout patients with a positive family history of CHD should also take precautionary measures to avoid heart complications. Nation-wide large-scale analyses considering rural/urban disparities are recommended for more in-depth investigations of influencing factors to prevent cardiovascular complications among patients with gout since diversity in demographic, lifestyle and environmental factors may influence the overall impact. Future analysis with lipids as a continuous variable is also suggested for more accurate estimates.

## Figures and Tables

**Figure 3:**
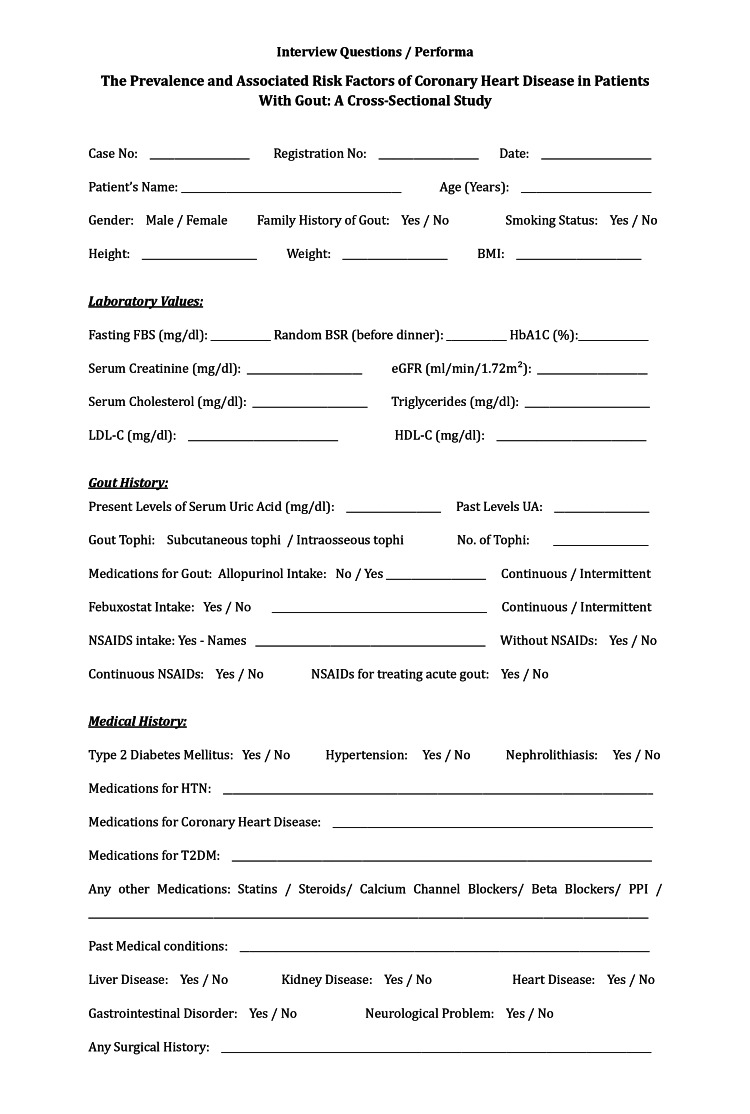
Interview questions - performa
